# Spontaneous Cure of Congenital Division of the Palatine Arch. Transactions of the Surgical Society

**Published:** 1868-04

**Authors:** 


### ARTICLE III.

*Spontaneous Cure of Congenital Division of the Palatine Arch. Transactions of the Surgical Society.*

(Translated from the French.)

M. Trélat brought forward a man 48 or 50 years old with an odd and interesting peculiarity, of which there exists, so far as he knows, no record in medical science. This patient came to consult him for a painful crepitation of the tendons. On hearing him speak, M. Trélat was struck by the intonation of his voice, closely resembling that of a person who had undergone a successful operation of staphylorrhaphy.

Examining the buccal cavity, M. Trélat saw that the soft palate had a peculiar appearance analogous to that which this organ presents after a successful operation of staphylorrhaphy. Still the patient, when interrogated repeatedly and in different ways, invariably insisted that he had never undergone the slightest operation. He only remembers that during his childhood, a surgeon had repeatedly urged his relatives to send him to Paris to be operated upon ; but this advice had not been followed. According to his statement, the cure of his congenital infirmity must have been anterior to the age of thirteen years, when he was bound out as an apprentice.

M. Trélat asked if his colleagues knew any facts of this nature, that is to say, cases of congenital fissure of the *velum palati* spontaneously healed, without a surgical operation : for his part he knew of none.

Examination of the *velum palati* of this man establishes an opinion some time since expressed by M. Trélat, viz.: that the difficulty which patients successfully operated on by staphylorrhaphy, experience in pronouncing certain words and certain letters, is caused by the shortness of the *velum palati*, which cannot come in contact with the parietes of the pharynx, whether this shortness depends on the incomplete union or the insufficiency of the *velum palati* itself, or whether it arises from insufficiency or irregularity of the developement of the palatine arch. This, instead of presenting its usual form is more or less hollowed out or contracted, offering thus the intermediate stages between simple division of the *velum pendulum palati* and simultaneous division of the velum and the palatine arch. It is one of the varieties of defective formation which is observed in this patient. Besides, the reunion of the divided velum, whether natural or artificial, is not absolutely perfect; a slight emargination can be detected at the point. This is why the voice and the articulation in this man are not all that could be desired, and remain somewhat defective. The patient has, at no period of his life, had any disease capable of inducing perforation of the palatine arch.

M. Demarquay has had occasion to see a person who bore on his upper lip the most evident marks apparently of an operation for hare-lip, which had, nevertheless, never been performed. He was born with this scar, an incontestible evidence of the spontaneous cure of a hare-lip during intra-uterine life. This man had a child with hare-lip complicated with division of the palatine arch. As an example of the hereditary nature of this vice of conformation, M. Demarquay knows now of three children so affected who belong to the same family.

M. Verneuil knew, as every one else of the spontoneous cure of hare-lip during intra-uterine life, examples of which are not rare. He has also seen a very curious case which proves that accidental hare-lip is equally capable of cure during intra-uterine life. He has seen and deposited in the Musée Dupuytren, a fœtus whose upper lip had a hare-lip scar, while on one side of the lower lip was seen a cicatrix exactly similar, which it was difficult to regard as any thing else than an accidental traumatic division spontaneously healed.

MM. Cloquet and Guersant say that they too have seen cases of hare-lip in which nature performed the entire cure without the intervention of art.

M. Trélat did not ask his colleagues if they knew cases of spontaneous cure of hare-lip; which are not very rare and of which he himself has collected a number. What he asks for is a case similar to that of the patient subjected to the inspection of his colleagues, which is an instance of spontaneous cure, after birth, of a congenital fissure of the palate, without surgical intervention. He believes this fact unique in science, and it is evident that none of his colleagues has seen any thing like it.

As to the facts concerning the hereditary nature of hare-lip of which M. Demarquay has spoken, they are not rare. M. Trélat knows of cases in which hereditary influence is evident in three and even five consecutive generations. Sometimes the disease leaps over one or more generations, just as other diseases thus transmissable are known to do.

M. Trélat showed his colleagues a drawing representing the velum and palatine arch of the patient who is the subject of this curious observation. The individual himself was then brought into the hall and submitted to the inspection of the members of the Surgical Society. He was then subjected to the test of reading aloud, and a defect of pronunciation similar to that observed in patients who have undergone staphylorrhaphy, was easily detected.

